# Off the RADAR; questions regarding the trial protocol of a randomised controlled trial of antipsychotic reduction and discontinuation

**DOI:** 10.1177/02698811221144633

**Published:** 2023-01-12

**Authors:** Sameer Jauhar, Paolo Fusar-Poli, David Foreman

**Affiliations:** 1 Department of Psychological Medicine, IoPPN, King’s College, London, UK; 2 Department of Psyhcosis Studies, IoPPN, King’s College, London, UK; 3 Department Child and Adolescent Psychiatry, King’s College, London, UK

**Keywords:** Antipsychotics, randomised controlled trial, schizophrenia

## Abstract

The randomised controlled trial of antipsychotic reduction and discontinuation addresses essential questions with regard to continued use of antipsychotics in schizophrenia, pertaining to social functioning and continued antipsychotic use. However, significant methodological issues stated in the trial protocol have the potential to confound interpretation of any findings. These include use of a non-blinded outcome measure, treatment as usual comparator and possible sample size issues.

We read with interest the trial protocol for Research into Antipsychotic Discontinuation and Reduction (RADAR), a randomised controlled trial (RCT) of gradual antipsychotic reduction and discontinuation (Moncrieff et al., 2019), and look forward to publication of its findings.

In our opinion, two design issues may affect interpretation of trial results,

## Use of treatment as usual comparator, non-blinded primary outcome

The study design is an open-label RCT, with treatment as usual (TAU) as comparator, the primary outcome being social functioning, measured by the social functioning scale (SFS) (Birchwood et al., 1990). This asks questions of the participant, with no inference made by the investigator. Participants are randomised to either antipsychotic reduction/discontinuation or TAU (maintenance treatment). In the intervention arm they appear to see a study psychiatrist, who one presumes may have contact with their treating team.

A non-blinded primary outcome measure (the SFS) is used despite the fact that clinician-assessed (and therefore potentially blinded) scales such as the Personal and Social Performance are available and used in clinical trials of antipsychotic medication (Kahn et al., 2018). Although the assessor may be blinded, the SFS score itself will be non-blinded as it relies wholly on subjective report of the participant, who will know if they are in the intervention arm or not.

In RADAR the intervention arm sees their treating team every 2 months and will therefore receive more input than TAU.

Three sources of bias (placebo effect, demand characteristics and investigator allegiance) could thus affect SFS scores. Unlike regression to the mean and random variation, the placebo effect is a non-specific psychological response to an intervention, with its own mechanisms. It is invoked by stimuli such as differential attention and expectation, good quality relationships and can generate statistically significant differences (Leucht et al., 2017; Wager and Atlas, 2015).

As participants are aware of what the researchers are investigating, what their intended findings are and how participants are expected to behave (McCambridge et al., 2012; Orne, 2017), they may modify their behaviour, in an attempt to be “good subjects”, as recognised in the Psychology literature for 60 years. This could be exacerbated by allegiance bias, where the study investigators might have a potential interest in the intervention.

Therefore, the study design makes it impossible to tell if any change in SFS is due to intervention (antipsychotic discontinuation or reduction), or non-blinded outcome measure and potential effects on the intervention arm, which have not been controlled for.

Whilst it may have been difficult to incorporate the ideal psychological placebo (or antipsychotic placebo), contacts with the community mental health team could have been matched, as could contact with the study psychiatrist. Moreover, there may be potential nocebo effects within the TAU group.

## Power calculations

Adequate power is essential for interpretation of study results. As well as theoretical issues (Button et al., 2013) practical studies have shown low power exaggerates effects of bias (Kossmeier et al., 2020). As written, we could not understand which power calculation is informing the sample size calculation, specifically whether the authors intend both a superiority or non-inferiority analysis, and what assumptions were made. In the protocol they describe a sample size calculation estimating *N* = 134–206 based on a 4–5-point Minimal Clinically Important Difference (MCID) for SFS but do not make clear how they derived this, though the use of a MCID is sensitive to method choice (Woaye-Hune et al., 2020). The authors reference three studies to indicate sensitivity to change sufficient to identify ‘good and poor outcome’ (Hellvin et al., 2010; Jaracz et al., 2015; Leeson et al., 2012). Only one cited study measured good and poor outcome (Jaracz et al., 2015). The other cited studies do not demonstrate a relevant significant difference in SFS. One was a validation of a Norwegian version of the SFS, with comparison between bipolar disorder, schizophrenia and healthy controls, showing a difference between groups (Hellvin et al., 2010), the other was in people with schizophrenia who used and did not use cannabis, where no difference was found at follow-up (Leeson et al., 2012). They also cite four studies as showing a difference in SFS between antipsychotics (Ciudad et al., 2006; Jang et al., 2011; Koshikawa et al., 2016; Sheridan et al., 2015); only two examined antipsychotics (Ciudad et al., 2006; Koshikawa et al., 2016). They state the scale differentiates those with short and long duration of untreated psychosis, though no difference was seen in total score (Barnes et al., 2008). The authors also appear to have used Mean and Standard Deviation values for sample size calculation from different study populations, with no clear explanation given.

There is a non-inferiority sample size calculation for severe adverse events, reporting *N* = 402 for a 10% margin. With recruitment completed, their final sample size is *N* = 253 (University College, London, 2020).

Current European guidance recommends significant adverse events be given priority in power calculations (Anonymous, 2018: 1). The figure of 253 is less than either their own non-inferiority estimate or recommended range (300–600) in the guidance. According to the published protocol, summary review of serious adverse effects will only take place at 3 months. As the intention is to reduce medication every 2 months for around a 12-month period, the ability to detect adverse events before study completion appears limited.

The study design has two unusual features, both of which can affect power. First, comparing two maintenance regimes, one reducing and one not. For both regimes, the treating clinician will be making changes based on the participants’ clinical presentation. This will affect outcome measures in RADAR and imposes a within-participant correlation on RADAR’s repeated measures of progress.

The authors are, quite reasonably, anticipating their postulated improvement in social functioning might be associated with deterioration in symptomatology, as a result of medication reduction. However, and irrespective of clinical significance of these symptoms, studies they cite identify associations between symptom scores and SFS scores. Thus, the study design imposes a negative bias on SFS scores, relative to studies used for their power calculations (none of which involved antipsychotic reduction or withdrawal). They will thus potentially over-estimate differences that RADAR might expect, leading to potential power over-estimation. One study they cite (Hellvin et al., 2010) reports the SFS correlates −0.12 (non-significant) with positive PANSS, and −0.28 for negative PANSS. The explained variance (square of the correlation coefficients) might significantly affect power and sample size needed.

We undertook our own power calculations, to test these theoretical concerns. We estimated power and confidence intervals for their final sample size (253), together with sample size for a power of 0.9 as per their protocol. Despite the odd number of participants, we assumed a 1:1 intervention: control ratio, as this was their original design, and we could not be sure which arm the odd participant was assigned to. We compared uncorrelated and correlated within-participant measurements, using the online package GLIMMPSE, which can model within-subjects correlation using Linear Exponents of type 1 AutoRegressive (LEAR) models, with parameters for both initial correlation and subsequent decay. We examined the treatment effect assuming a superiority design, a 5-point mean difference at 24 months, smoothly increasing, and the authors’ 8.8 SD in SFS, with 0.05 probability of type 1 error. We used both the default LEAR parameters recommended in GLIMMPSE, and parameters assuming the initial correlation would be the same as for repeat reliability of the SFS (0.78) with the maximum decay recommended by the authors of GLIMMPSE as ‘compatible with biological systems’. We believe this covers the plausible range of within-subject correlations and decay over study duration. We considered unadjusted differences, and notional 10% shrinkage in mean difference to account for the overlap with worsening symptomatology noted above. We estimated confidence intervals for power based on their 5-point difference. We tabulate our results below (Table 1). The first column gives relevant power for RADAR’s sample size, the second and third columns give values of lower (0.025) and upper (0.975) bounds of the values’ 95% confidence intervals. The fourth column (assuming equal group sizes) gives group sample size necessary to achieve 0.9 power under different LEAR parameterisations, and the last two columns give postulated time-dependent intercorrelations in within-subject scores and their decay.

**Table 1.** Sample size calculation.

Power for RADAR sample size	0.025 Confidence Interval	0.975 Confidence Interval	Group sample size to achieve 0.9 power	LEAR adjustment	
				Correlation	Decay
0.99	0.66	1	138	0	0
0.75	0.1	1	386	0.5	0.3
0.60	0.05	0.99	538	0.78	0.5
10% Shrinkage in mean difference					
0.99	0.34	1	140	0	0
0.74	0.05	1	392	0.5	0.3
0.59	0.05	0.99	546	0.78	0.5

LEAR: Linear Exponents of type 1 AutoRegressive.

The table shows that, while shrinkage had less effect, the uncertainty in their estimated power increases sharply as the LEAR parameters increase, as do the predicted sample sizes, while power decreases. Therefore, the sample size of 253 is possibly insufficient to detect the MCID identified, given the study design. It is well-understood that interpretation of negative findings is impaired in an underpowered trial: here, negative findings could relate to symptomatic deterioration or adverse events. However, lack of power also impairs the interpretation of positive findings. RCTs do not compare probability distributions, but alternative models, whose relative likelihood may be assessed by the Bayes factor (Dienes and Mclatchie, 2018). A criterion for preferring H_1_ of *p* < 0.05 for H_0_ translates to a Bayes factor of about three, that is, the model generating H_1_ is about three times as likely as that generating H_0_. Thus, the chances of being struck by ‘survivor’s curse’ in an underpowered design is much greater than the 1:20 the typical *p* < 0.05 cutoff suggests.

## Conclusion

This is a much-needed trial, though the design (use of essentially a self-report outcome measure, TAU comparator with resultant effects on primary outcome measure) and issues with power makes interpretation of findings difficult.
